# -308G/A polymorphism of tumor necrosis factor alpha (*TNF-α*) gene and metabolic syndrome susceptibility: a meta-analysis

**DOI:** 10.1038/s41598-021-83321-x

**Published:** 2021-02-15

**Authors:** Dong Wang, Liqun He, Xiaotian Zhang

**Affiliations:** 1 Department of Cardiology, Wuhan No. 1 Hospital, Wuhan Hospital of Traditional Chinese and Western Medicine, 215# Zhongshan Ave., Wuhan, 430022 People’s Republic of China; 2 Hubei Provincial Key Laboratory for Applied Toxicology, Hubei Provincial Center for Disease Control and Prevention, 35# Zhou Daoquan North Road, Wuhan, 430079 People’s Republic of China

**Keywords:** Endocrinology, Genetic association study, Medical genetics

## Abstract

Many studies tried to assess the relationship between -308G/A polymorphism of tumor necrosis factor alpha (*TNF-α*) gene and risk of metabolic syndrome (MS), but their results were contradictory. This meta-analysis aimed to precisely evaluate this association. A systematic literature search was performed in Pubmed database and WanFang Med Online, STATA software 14.0 was used for the meta-analysis. Eleven independent studies containing 3277 cases and 3312 controls were included in our meta-analysis. In overall analysis, significant association was found between -308G/A polymorphism of *TNF-α* and MS in both allele model (OR 1.47, 95% CI 1.09–1.98, P 0.013) and dominant model (OR 1.77, 95% CI 1.21–2.58, P 0.003). In the subgroup analysis, the A allele was associated with increased risk of MS in Asia group (allele model: OR 1.82 95% CI 1.31–2.53, P < 0.001; dominant model: OR 2.30, 95% CI 1.64–3.21 P < 0.001; homozygous model: OR 2.29, 95% CI 1.31–4.01, P 0.004), and decreased risk of MS in Europe group (dominant model: OR 0.83, 95% CI 0.70–0.99, P < 0.001; recessive model: OR 0.51, 95% CI 0.28–0.92, P 0.025; homozygous model: OR 0.49 95% CI 0.27–0.89, P 0.02). The A allele also appeared to linked to increased risk of MS in CDS group and IDF groups. No significant association was observed in NCEPATPIII group. Our results suggested that -308G/A of *TNF-α* gene was a risk factor for MS, but it may played different roles in different ethnics, further studies with larger sample size and more other ethnics should be performed to confirm our conclusions.

## Introduction

Metabolic syndrome (MS) is a multi-component disease characterized by the combination of a series of clinical and biochemical metabolic disorders including insulin resistance, elevated plasma triglyceride levels, decreased HDL-c, hypertension, hyperglycaemia and abdominal obesity. In 1998, the first criteria of MS was made by World Health Organization (WHO)^1^, since then many international organizations and expert groups, such as European Group for the study of Insulin Resistance (EGIR)^2^, the International Diabetes Federation (IDF)^3^, the National Cholesterol Education Program Adult Treatment Panel III (NCEP:ATPIII)^4^, the American Association of Clinical Endocrinology (AACE)^5^, Chinese Diabetes Society^6^, have defined MS using different parameters. Among those definitions, the ones made by NCEP:ATPIII and IDF were the most widely used currently. The morbidity of MS is increasing around the world, which makes MS a huge public health burden for many countries^3,7–9^. Many factors are involved in the development of MS. On one hand, environmental factors such as physical inactivity and improper eating habits are essential determinants for MS. On the other hand, genetic factors also play vital roles in the development of MS.

Tumor necrosis factor alpha (*TNF-α*) is a multifunctional pro-inflammatory cytokine with both beneficial and destructive functions for human body produced by various kinds of cells such as macrophages, fibroblasts, epithelial cells, adipocyte^10^. As we known, *TNF-α* could regulate numerous inflammatory and autoimmune processes, and participates in many life activities such as apoptosis, differentiation and cell recruitment^11^. Prior studies also demonstrated that *TNF-α* combined with other types of pro-inflammatory cytokines contributed to the development of type II diabetes, obesity and obesity-induced insulin resistance, which suggested *TNF-α* played as an essential role in the development of MS^12–15^. *TNF-α* gene is located at 6p21.1–21.3 chromosomal region, which is near to major histocompatibility complex. Wilson et al. firstly identified a G to A variant at 308 upstream of *TNF-α* gene promoter region in 1992^16^. Subsequent studies has shown that -308G/A had great impact on the transcription activation of *TNF-α* gene and was relevant with the increased plasma *TNF-α* plasma levels^17^. Observational studies supported -308G/A of *TNF-α* gene was associated with the components of MS such as hypertension and insulin resistance^18^. Many studies containing different ethnic subjects tried to reveal the relationship between -308G/A of *TNF-α* gene and MS susceptibility, however, those results were inconsistent.

In this study, we performed a meta-analysis to assess the accurate impact of the -308G/A polymorphism of *TNF-α* gene promoter on the MS risk.

## Methods

### Literature search

A systemic literature search was performed in Pubmed and Wanfang Online database to identify all the potential studies that involved the association between -308G/A polymorphism of *TNF-α* gene and MS susceptibility. Following key words were used for our literature search: (“tumor necrosis factor alpha” OR “*TNF-α*”) AND (polymorphism OR mutation OR variant) AND (metabolic syndrome OR MetS OR MS). The language was restricted to English and Chinese, and the search process was completed on April 6th 2020.

### Inclusion criteria

The following inclusion criteria should be met when the studies were included in this meta-analysis: (1) studies should examine the association between -308G/A polymorphism of *TNF-α* gene and MS susceptibility; (2) studies should be case–control designed or cohort designed; (3) studies should provide enough data to calculate the odds ratios (ORs) and the corresponding 95% confidence intervals (CIs).

### Data extraction and quality assessment

Two authors independently extracted the following information from each study: the first authors, the years of publication, the demographic information of each study, the numbers of case and control groups, and the frequencies of the three types of genotypes in both cases and controls. Newcastle–Ottawa quality assessment scale was used for the quality assessment of each research^19^.

### Statistical analysis

We calculated the pooled ORs and corresponding 95%CIs to assess the strength of the association between -308G/A polymorphism of *TNF-α* gene and MS susceptibility. Pooled ORs and 95% CIs were performed under four genetic models: allele model (A vs.G), dominant model (AA + GA vs. GG), recessive model (AA vs. GG + GA), and homozygous model (AA vs. GG), respectively. We used random effect models to calculate all the pooled ORs and corresponding 95%CIs. The Z tests were used to assess the significance of the ORs. Hardy–Weinberg equilibrium (HWE) was tested using Chi-square test in the control groups. Heterogeneity between the studies included in this meta-analysis was evaluated using χ^2^ based Q tests and I-square (I^2^) tests. We defined low, moderate, and high degrees of heterogeneity when the values of I^2^ were 25%, 50%, and 75%, respectively. We carried out sensitive analysis to assess the impact of each study on the pooled ORs and 95% CIs. The Begg’s and Egger’s tests were used to evaluate the publication bias. All tests were two-sided, and a value < 0.05 was considered as statistically significant. STATA software (version 14.0; State Corporation, College Station, TX, USA) was used to perform all the statistical tests in this meta-analysis.

## Results

### Study characteristics

**Figure 1:**
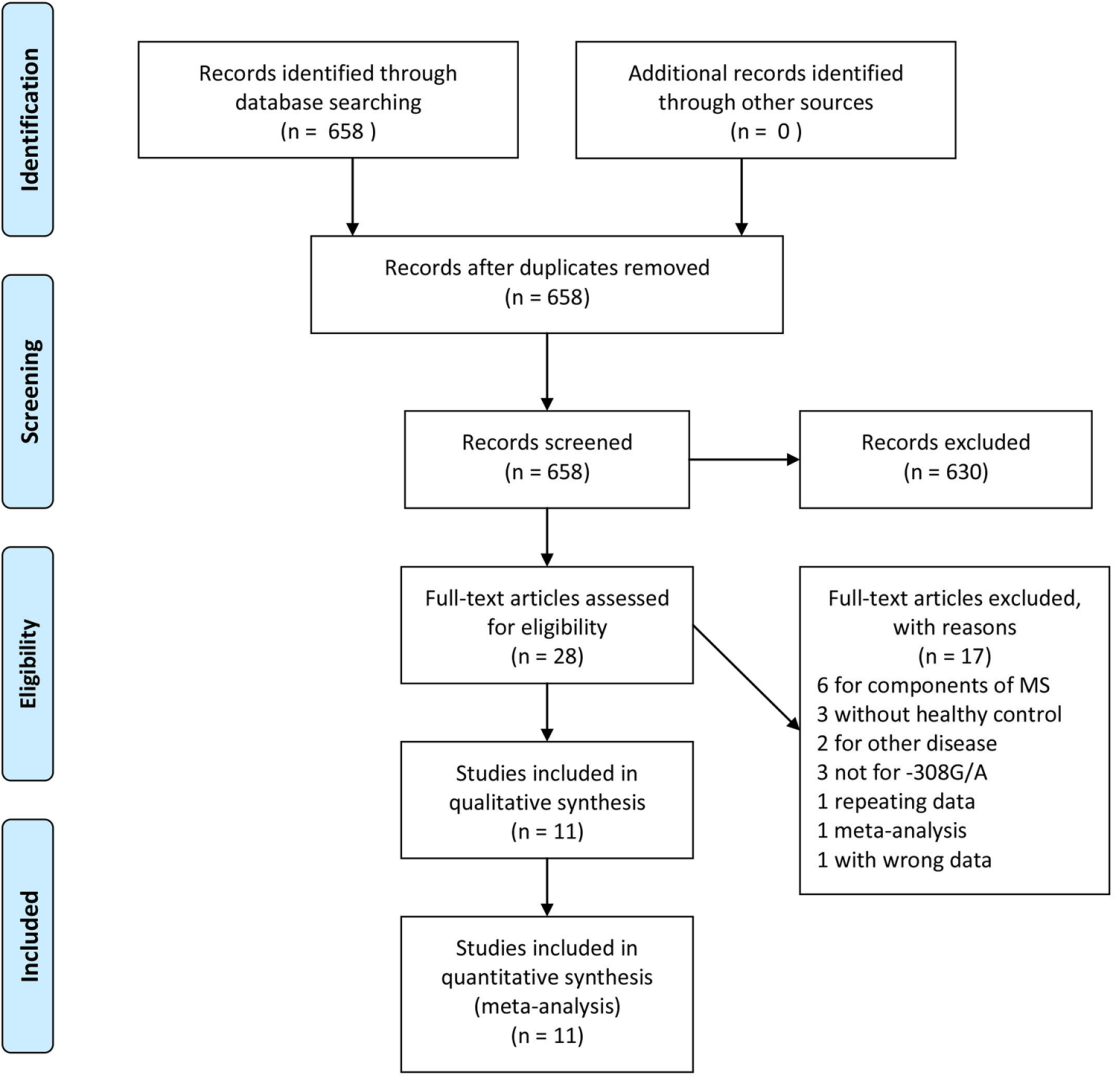
Flow chart of research and selection process.

Depending on the literature search strategy mentioned above, 658 articles were initially identified in the Pubmed database and Wanfang Med Online. The sift process was shown in Fig. 1. Briefly, 630 articles were excluded after reviewing their titles and abstracts. Of the remaining 28 articles, 17 ones were excluded for the following reasons: six studies were performed for the association between -308G/A polymorphism of *TNF-α* gene and the components of MS, three studies did not have healthy controls, three studies were for other polymorphism sties of *TNF-α* gene, two studies were for other disorders, one study with repeating data, one study was meta-analysis, one study with wrong data. Finally, eleven studies containing 3277 cases and 3312 controls were included in our meta-analysis. Eight of the eleven studies were published in English, three ones were in Chinese. The publication time of the eleven studies ranged from 2005 to 2019. There are three types of diagnostic criteria were used for the diagnosis of MS, five studies diagnosed MS using the National Cholesterol Education Program Adult Treatment Panel III (NCEPATP III) definition, four studies adopted International Diabetes Federation (IDF) definition, two studies recruit their subjects using the definition made by Chinese Diabetes Society (CDS). Four different kinds of genotyping methods existed, the numbers of studies used restriction fragment length polymorphism (RFLP) was eight. Golden Gate Assay, Taqman probe, melting curves were respectively used in the remaining three studies. All the studies included in our studies were in HWE (P > 0.05). The general characteristics of the eleven studies and the frequencies of different genotypes extracted from each study were shown in the Table 1.

**Table 1:** General characteristics of studies included in the meta-analysis.

Author	Year	Language	Country	Diagnostic criteria	Source of control	Sample size (case/control)	Method	Genotype										HWE	NOS	Ref
								Case					Control							
								G	A	GG	GA	AA	G	A	GG	GA	AA			
Bu	2014	English	China	IDF	HB	200/200	RFLP	351	49	162	27	11	384	16	188	8	4	Yes	7	20
Uzma	2019	English	Pakistan	IDF	HB	224/200	RFLP	301	147	100	101	23	280	120	102	76	22	Yes	8	21
Vani	2012	English	India	NCEPATP III	HB	269/272	RFLP	429	109	169	91	9	502	42	230	42	0	Yes	6	22
Zhao	2013	Chinese	China	NCEPATP III	HB	600/600	Taqman	1083	117	488	107	5	1142	58	543	56	1	Yes	7	23
Catherine	2010	English	France	NCEPATP III	PB	877/877	Golden Gate Assay	1549	205	683	183	11	1494	260	635	224	18	Yes	6	24
Ranbir	2012	English	India	NCEPATP III	HB	245/321	RFLP	236	254	11	214	20	343	299	56	231	34	Yes	8	25
Aline	2005	English	France	NCEPATP III	PB	601/594	RFLP	496	79	198	73	3	1409	269	599	211	29	Yes	7	26
Gong	2016	Chinese	China	CDS	HB	119/60	RFLP	204	34	89	26	4	111	9	51	9	0	Yes	7	27
Fan	2019	Chinese	China	CDS	HB	48/60	RFLP	81	15	35	11	2	113	7	53	7	0	Yes	8	28
Seyed	2015	English	Iran	IDF	HB	94/128	RFLP	122	66	41	40	20	168	88	60	48	20	Yes	7	29
Szkup	2018	English	Poland	IDF	PB	118/298	Melting curves	201	35	85	31	2	515	81	226	63	9	Yes	8	30

*HB* hospital based, *PB* population based, *IDF* the International Diabetes Federation, *NCEPATP III* the National Cholesterol Education Program Adult Treatment Panel III, *HWE* Hardy–Weinberg Equilibrium, *CDS* Chinese Diabetes Society, *RFLP* restriction fragment length polymorphism, *NOS* Newcastle–Ottawa Score.

### Meta-analysis

**Figure 2:**
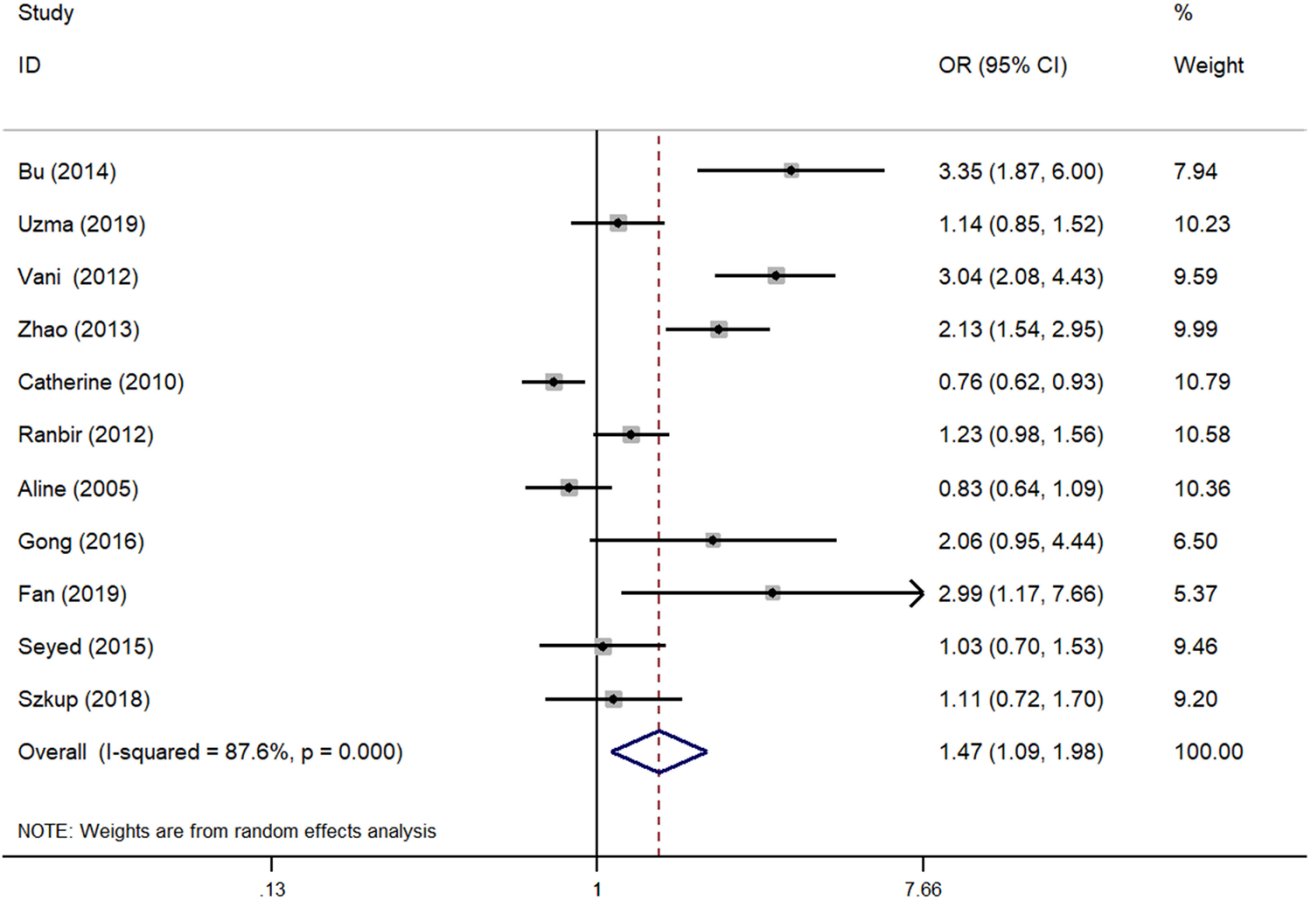
Forest plot of the association between -308G/A polymorphism of *TNF-α* gene promoter and MS risk under allele model. *OR* odds ratio, *CI* confidence interval.

We evaluated the association between -308G/A polymorphism of *TNF-α* gene and MS susceptibility in four genetic models. Overall, significant association was found in both allele model (OR 1.47, 95% CI 1.09–1.98, P 0.013) (Fig. 2) and dominant model (OR 1.77, 95% CI 1.21–2.58, P 0.003). No significant association was observed in recessive model (OR 1.08, 95% CI 0.67–1.74, P 0.744) and homozygous model (OR 1.50, 95% CI 0.84–2.70, P 0.174).

**Figure 3:**
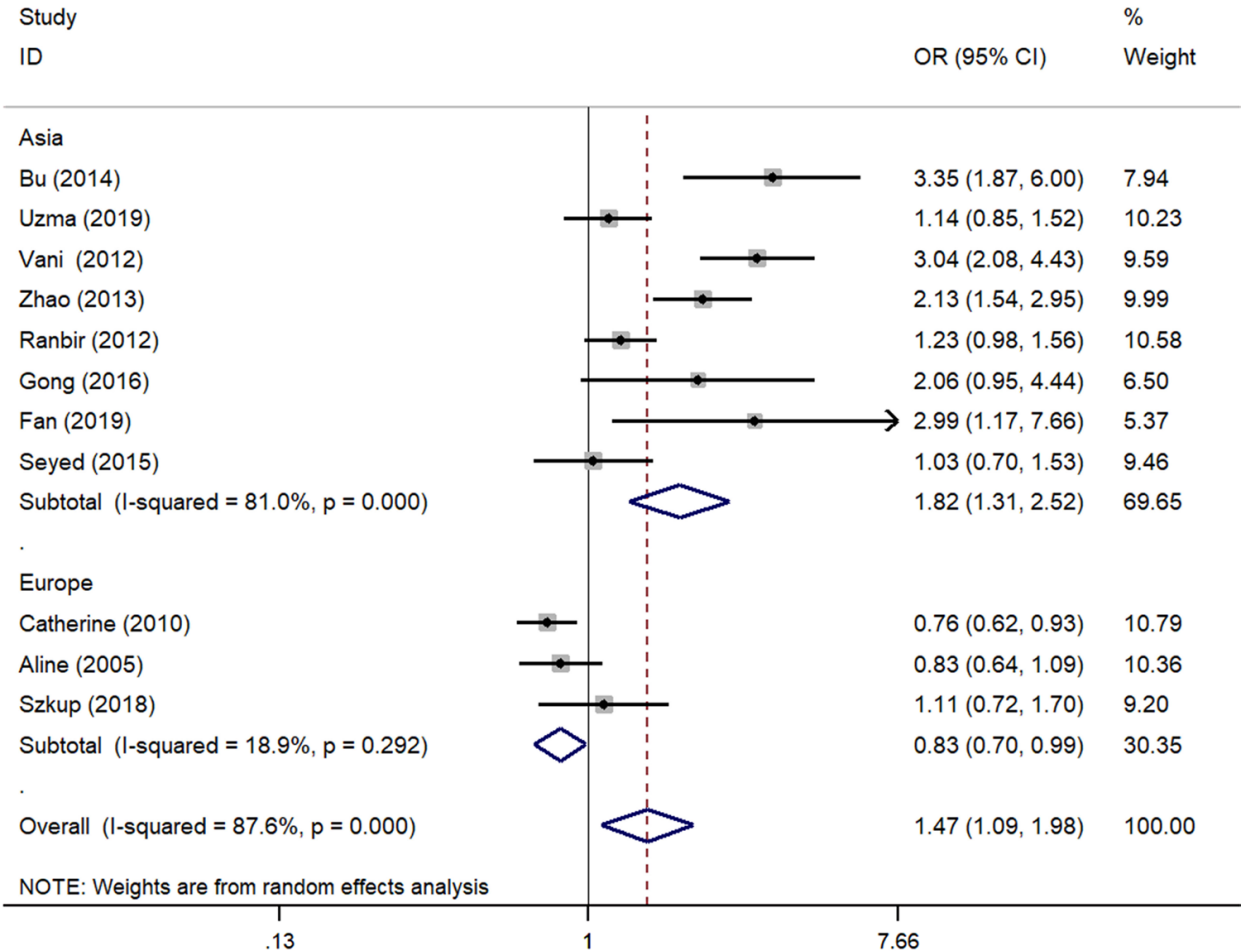
Subgroup analysis stratified by regions under allele model. *OR* odds ratio, *CI* confidence interval.

**Figure 4:**
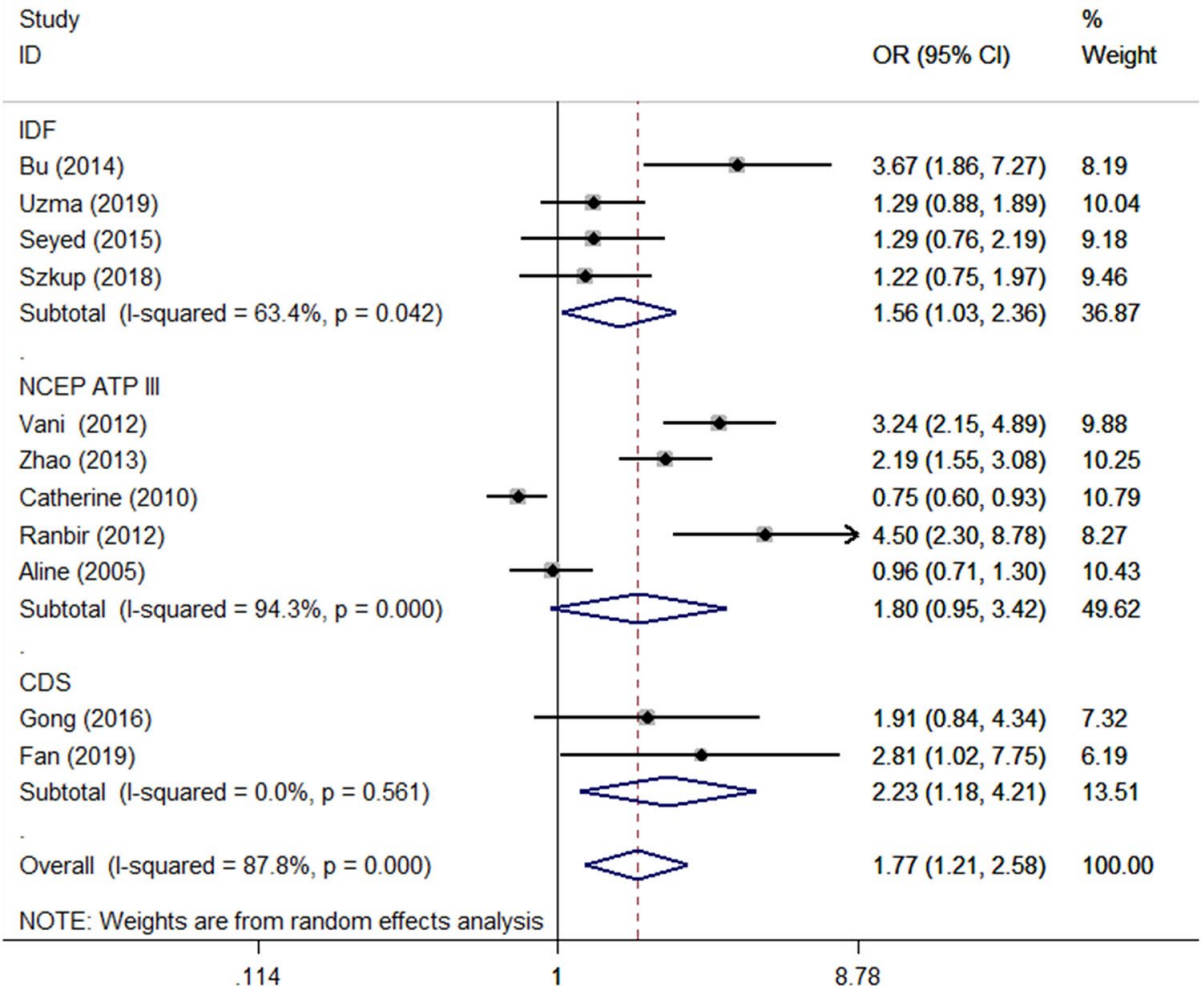
Subgroup analysis stratified by criteria under dominant model. *OR* odds ratio, *CI* confidence interval. *IDF* the International Diabetes Federation, *NCEPATP III *the National Cholesterol Education Program Adult Treatment Panel III; *CDS* Chinese Diabetes Society.

In the subgroup analysis performed by the regions of the studies, A allele showed increased risk of MS in Asia group (allele model: OR 1.82 95% CI 1.31–2.53, P < 0.001; dominant model: OR 2.30, 95% CI 1.64–3.21 P < 0.001; homozygous model: OR 2.29, 95% CI 1.31–4.01, P 0.004). Inversely, the A allele showed decreased risk of MS in Europe group (dominant model: OR 0.83, 95% CI 0.70–0.99, P < 0.001; recessive model: OR 0.51, 95% CI 0.28–0.92, P 0.025; homozygous model: OR 0.49 95% CI 0.27–0.89, P 0.02) (Fig. 3). When subgroup analysis was stratified by diagnostic criteria, the A allele showed increased risk of MS in CDS group (allele model: OR 2.39, 95% CI 1.31–4.34, P 0.004; dominant model: OR 2.23, 95% CI 1.18–4.21, P 0.014) and IDF groups (dominant model: OR 1.56, 95% CI 1.03–2.36, P 0.038) (Fig. 4). No significant association was observed in NCEP ATP III group. When stratified by the source of control, the results were the same as the ones stratified by regions of the studies (Fig. 4; Table 2).

**Table 2:** The association between -308G/A polymorphism of *TNF-α* gene and MS susceptibility.

Group	No. of studies	Case	Control	Pool OR	95% CI	P	I^2^	P_h_	Begg' test	Egger' test
**Allele model (A vs. G)**										
Overall	11	3277	3312	1.47	1.09–1.98	0.013	80.93%	< 0.001	0.061	0.032
Region										
Asia	8	1799	1841	1.82	1.31–2.53	< 0.001	81.00%	< 0.001		
Europe	3	1696	1796	0.83	0.70–0.99	0.033	18.90%	0.0048		
Diagnostic criteria										
IDF	4	636	826	1.37	0.90–2.01	0.138	76.30%	0.006		
NCEP ATP III	5	2592	2664	1.36	0.85–2.18	0.2	93.50%	< 0.001		
CDS	2	167	120	2.39	1.31–4.34	0.004	0.00%	0.546		
**Dominant model (AA + GA vs GG)**										
Overall	11	3277	3312	1.77	1.21–2.58	0.003	82.19%	< 0.001	0.161	0.017
Region										
Asia	8	1799	1841	2.3	1.64–3.21	< 0.001	67.70%	0.003		
Europe	3	1696	1796	0.9	0.69–1.17	0.426	51.80%	0.126		
Diagnostic criteria										
IDF	4	636	826	1.56	1.03–2.36	0.038	63.40%	0.042		
NCEP ATP III	5	2592	2664	1.8	0.95–3.42	0.72	94.30%	< 0.001		
CDS	2	167	120	2.23	1.18–4.21	0.014	0.00%	0.561		
**Recessive model (AA vs GG + GA)**										
Overall	11	3277	3312	1.08	0.67–1.74	0.744	49.40%	0.032	0.161	0.077
Region										
Asia	8	1799	1841	1.52	0.87–2.67	0.14	47.30%	0.066		
Europe	3	1696	1796	0.51	0.28–0.92	0.025	0.00%	0.636		
Diagnostic criteria										
IDF	4	636	826	1.19	0.73–1.95	0.48	22.10%	0.278		
NCEP ATP III	5	2592	2664	0.91	0.39–2.11	0.821	63.10%	0.028		
CDS	2	167	120	5.5	0.66–45.81	0.115	0.00%	0.028		
**Homozygous model (AA vs GG)**										
Overall	11	3277	3312	1.5	0.84–2.70	0.174	61.00%	0.004	0.533	0.169
Region										
Asia	8	1799	1841	2.29	1.31–4.01	0.004	37.20%	0.132		
Europe	3	1696	1796	0.49	0.27–0.89	0.02	0.00%	0.686		
Diagnostic criteria										
IDF	4	636	826	1.34	0.81–2.21	0.256	19.70%	0.291		
NCEP ATP III	5	2592	2664	1.65	0.48–5.75	0.428	79.50%	0.001		
CDS	2	167	120	6.2	0.74–51.78	0.092	0.00%	0.862		

*IDF* the International Diabetes Federation, *NCEPATP III* the National Cholesterol Education Program Adult Treatment Panel III, *CDS* Chinese Diabetes Society, *OR* odds ratio, *CI* confidence interval.

Results of tests for heterogeneity were also shown in Table 2. High degree heterogeneity was shown in allele model (I^2^ 80.93%, P_h_ < 0.001) and dominant model (I^2^ 82.19%, P_h_ < 0.001). Low and moderate degree heterogeneity was found in recessive model (I^2^ 49.94%, P_h_ 0.032) homozygous model (I^2^ 61.00%, P_h_ 0.004), respectively. Sensitive analysis was performed by omitting one study at a time and then the ORs and 95% CI of the remaining studies were recalculated. In this study, the pooled ORs and the 95% CI calculated after excluding single study did not show violent fluctuation compared with the value of overall analysis, suggesting the results of our study were stable. Publication bias analysis was performed using Begg’s and Egger’s tests in all the four genetic models. As shown in Table 2, all the P values were > 0.05 except the Egger’s test in dominant model (P = 0.017).

## Discussion

Prior studies have revealed chronic inflammation may be one of most important promoting factors for the development of MS^31–35^. *TNF-α* secreted by M1 pro-inflammatory adipose tissue macrophages is one of the most important pro-inflammatory mediators in various organs of human body and could initiate the NF-κB and JNK pathways involved in the insulin resistance and the apoptosis of the β cells within the pancreatic islets^36–41^, which suggested *TNF-α* may play a central role in the development of MS. Many case–control studies tried to assess the relationship between -308G/A polymorphism of *TNF-α* gene promoter and the risk of MS, however, their results were inconsistent and with low statistic power due to the small sample sizes of those studies.

In this study, we performed a meta-analysis to evaluate the relationship between -308G/A polymorphism of *TNF-α* gene promoter and MS risk. Eleven studies containing 3277 cases and 3312 controls were included totally. Our results revealed that the A allele increased 48%, 77% risk for MS in allele model and dominant model in overall study, respectively. In the subgroup analysis, it was of great interests for us to find opposite conclusions in the Asia and Europe subgroup. The A allele distinctly increased MS susceptibilities in allele model (OR 1.82, 95% CI 1.31–2.53, P < 0.001), dominant model (OR 2.30, 95% CI 1.64–3.21, P < 0.001), and homozygous model (OR 2.29, 95% CI 1.31–4.01, P 0.004) in Asia subgroups, while in Europe subgroup the A allele seemed to play a protective role for MS in allele model (OR 0.83, 95% CI 0.70–0.99, P 0.033), recessive model(OR 0.51, 95% CI 0.28–0.92, P 0.025) and homozygous model (OR 0.49, 95% CI 0.27–0.89, P 0.02). Our results showed the complexities for MS once again. Why did the same variant play antipodal roles of MS in different countries? MS is a kind of complex and polygenetic disorder. Different ethnic group may have entirely different genetic background. Silvia et al. performed a meta-analysis to assess the relationship between -308G/A polymorphism and the risk of MS components^18^. They found ethnicity was also an important factor to influence relationship between -308G/A polymorphism and hypertension, T2DM and obesity. Our study further demonstrated that -308G/A polymorphism could play different roles in the development of MS in different ethnicities on the base of Silvia et al. Some issues should be discussed when we explained this result: firstly, the three studies for Europe included were population-based, while the eight studies from Asia were hospital-based, whether the different methods to include the control populations by each study influence the results of the two subgroups still need to be consulted, even if the minor allele frequencies of A was near to each other between the two the control groups (0.173 in Asia group and 0.151 in Europe group). Secondly, as another risk factor, age also played vital roles in the development of MS, another meta-analysis performed by us revealed the rs9939609 of *FTO* gene was associated with MS in Chinese adults but not in Chinese children and adolescents^42^, that means time is needed in the interaction between genetic factors and environment. The subgroup analysis stratified in different age groups could not be performed in our study. Thirdly, in our study, only three studies in Europe were included the sample size was still limited, further studies with larger sample sizes especially from Europe should perform to confirm our conclusions.

The heterogeneity of meta-analysis usually comes from three aspects: clinical heterogeneity, statistical heterogeneity, and methodological heterogeneity. During the overall analysis, high degrees of heterogeneity was found in the allele model (I^2^ = 80.93%, P < 0.001) and dominant model (I^2^ = 82.19%, P < 0.001), while the heterogeneity in recessive model and homozygous model was low (I^2^ = 49.40%, P 0.032) and moderate (I^2^ = 61.00%, P 0.004). The results from overall analysis indicated that the different statistical methods could be a source of heterogeneity. In the subgroup analysis, we found the levels of heterogeneity were dramatically decreased in the Asia and Europe subgroups in all the four genetic models, suggesting that the ethnic may be an important source of heterogeneity. In the subgroup analysis stratified by the diagnostic criteria, lower levels of heterogeneity were found in IDF subgroups and no heterogeneity was found in CDS subgroup, the main heterogeneity came from the NCEP ATP III subgroups, which means diagnostic criteria should be another important source of heterogeneity.

Begg’s and Egger’s tests were performed to assess the publication bias tests, A P value 0.017 was found in the Egger’s test in dominant model. Although there were no statistical significance was found in the other Begg’s and Egger’s tests, potential publication bias may exist in our study. Because only eleven studies were included in our meta-analysis, limited number of studies may also contributed to the results of the tests for publication bias.

Our study still had several limitations should be mentioned: firstly, because we searched the articles only in Chinese and English, researches published in other languages were ignored, that may result in bias; secondly, other risk factors of MS such as physical inactivity, excessive food intake were not be considered in our meta-analysis; thirdly, the total sample size still limited in our research, and only studies from Asia and Europe were included, further studies with larger sample size and more other ethnics should be performed to confirm our conclusions.

To our knowledge, this is the first meta-analysis to assess the relationship between the -308G/A polymorphism of *TNF-α* gene promoter and the susceptibility of the MS. Our results suggested -308G/A polymorphism was significantly associated with increased MS susceptibility in overall analysis, in the further analysis a completely apposite result was found in the Asia and Europe subgroup. Our results illuminated the complex impact of -308G/A polymorphism of *TNF-α* gene promoter played on the MS susceptibility.
